# Measurement Invariance of the Internet Gaming Disorder Scale–Short-Form (IGDS9-SF) Between Australia, the USA, and the UK

**DOI:** 10.1007/s11469-017-9786-3

**Published:** 2017-07-24

**Authors:** Vasileios Stavropoulos, Charlotte Beard, Mark D. Griffiths, Tyrone Buleigh, Rapson Gomez, Halley M. Pontes

**Affiliations:** 1 University of Athens, Athens, Greece; 2 Federation University, Ballarat, Australia; 3 Palo Alto University, 1791 Arastradero Rd, Palo Alto, CA 94304 USA; 4 Nottingham Trent University, Nottingham, UK

**Keywords:** IGD, IGDS9-SF, Measurement invariance, Gamers, Internet gaming disorder, Gaming addiction

## Abstract

The Internet Gaming Disorder Scale–Short-Form (IGDS9-SF) is widely used to assess Internet Gaming Disorder behaviors. Investigating cultural limitations and implications in its applicability is imperative. One way to evaluate the cross-cultural feasibility of the measure is through measurement invariance analysis. The present study used Multigroup Confirmatory Factor Analysis (MGCFA) to examine the IGDS9-SF measurement invariance across gamers from Australia, the United States of America (USA), and the United Kingdom (UK). To accomplish this, 171 Australian, 463 USA, and 281 UK gamers completed the IGDS9-SF. Although results supported the one-factor structure of the IGD construct, they indicated cross-country variations in the strength of the relationships between the indicators and their respective factor (i.e., non-invariant loadings of items 1, 2, 5), and that the same scores may not always indicate the same level of IGD severity across the three groups (i.e., non-invariant intercepts for items 1, 5, 7, 9).

Internet Gaming Disorder (IGD) was suggested as a condition for further study in the most recent (fifth) edition of the *Diagnostic and Statistical Manual of Mental Disorders* (DSM-5) in 2013 by the American Psychiatric Association (American Psychiatric Association 2013). The phenomenon of IGD is broadly described as a form of persistent and recurrent involvement with video games, often leading to the decline of daily work and/or education activities (American Psychiatric Association 2013). However, problematic and addictive use of video games, as a wide-reaching behavior affecting children and adults, has facilitated research and promoted clinical awareness well before the introduction of IGD (Pontes, & Griffiths 2014; Stavropoulos, Kuss, Griffiths, Wilson, & Motti-Stefanidi 2017). In this context, a 2013 review of measurement instruments for *pathological gaming* (King, Haagsma, Delfabbro, Gradisar & Griffiths 2013) indicated the existence of 18 distinct instruments, with only one (PVP; Salguero and Moran 2002) covering all nine IGD diagnostic criteria as suggested by the American Psychiatric Association in the DSM-5 (American Psychiatric Association 2013). Given the heterogeneity and inconsistency with regard to most psychometric assessment tools previously identified, scholars have argued that this issue hindered the field of assessment of IGD (Pontes, 2016; Pontes & Griffiths 2014). In fact, these measurement inconsistency issues illustrated part of the “chaos and confusion” identified in the conceptualization and measurement of the IGD construct (Kuss, Griffiths, & Pontes 2017; p.1).

Consequently, it has been widely recognized that there is much to be desired in terms of international consensus on IGD in relation to conceptualization and comorbidities (Griffiths et al. 2016; Kind and Delfabbro 2014). Nevertheless, the introduction of the nine IGD diagnostic criteria by the DSM-5 (American Psychiatric Association 2013), largely based on the work of Tao et al. (2010), provided a diagnostic framework for Internet addiction based on previous research on pathological gambling and substance use disorder (Petry et al. 2015). In light of the existing methodological issues in the assessment of IGD, the need for a robust standardized measurement of IGD became a forefront issue in relevant research (Anderson, Steen & Stavropoulos 2016). To address this need, the Internet Gaming Disorder Scale–Short-Form (IGDS9-SF) (Pontes & Griffiths 2015) was developed as a brief screening tool for gaming addiction based on the nine criteria suggested by the DSM-5 (American Psychiatric Association 2013), and serves as a starting point for more standardized research in the field.

With research increasing globally into IGD across disciplines (i.e., clinical psychology, cognitive psychology, and human-computer interaction), it is has become paramount to evaluate the cross-cultural psychometric properties of an instrument such as the IGDS9-SF, with wide international implementation and use. Not only should a globally used measure demonstrate reliability and validity in one sample but also other underlying psychometric and scaling properties are important when using a single instrument in a variety of cultural contexts (Gomez & Rohner 2011; Gomez 2013). Measurement invariance (MI) refers to groups (i.e., cultures, genders) reporting the same observed scores when they exhibit the same level of the underlying trait (Gomez & Rohner 2011; Gomez 2013). As applied to the IGDS9-SF, MI would mean that for the groups (i.e., countries/cultures) being compared, the IGDS9-SF has the same measurement and scaling properties, allowing test scores on this measure to be psychometrically comparable. As support for MI is a prerequisite for meaningful group comparisons, it follows that if there is no support for MI across the groups being studied, then the results of such comparisons are confounded by differences in measurement and scaling properties for the IGDS9-SF across the groups. If there is weak or no support for invariance, then it is concluded that the groups in question cannot be justifiably compared in terms of the IGDS9-SF observed scores, as the same observed scores may not reflect the same levels of the underlying latent IGD construct being measured. Thus, if the IGDS9-SF is to be used across different countries (i.e., online and/or offline gaming populations), then it is necessary to demonstrate that the ratings of the IGDS9-SF across the countries being compared have MI, or at least, for researchers to know the nature of MI across these countries. Given the global nature of IGD behaviors and the cross-country comparability concerns already identified in the literature, this need (i.e., establishing MI) appears to be compelling (Kuss, Griffiths, & Pontes 2017).

One powerful method for testing MI is Multigroup Confirmatory Factor Analysis (MGCFA) (Gomez & Rohner 2011; Gomez 2013). This procedure aims to establish invariance of the items of a psychometric test across distinct groups considering the number of factors (i.e., configural invariance), item factor loadings (i.e., metric invariance), item intercepts and thresholds for continuous and categorical responses, respectively (i.e., scalar invariance), and error variances (Gomez & Rohner 2011; Gomez 2013). Support for *configural invariance* indicates that the same number of factors and the same patterns of free factor loadings hold across the groups. For example, the IGDS9-SF exhibits a one-factor model to represent the construct of IGD; therefore, configural invariance would support a one-factor structure across compared groups. Support for *metric invariance* indicates that the strength of the relationships between the items and their respective factors is equivalent across groups, and that across the groups, the items are measuring their relevant latent factors using the same metric scale. Support for *scalar invariance* indicates that for the same level of the latent trait, individuals across the groups will endorse the same observed levels (i.e., when observed scores are treated as continuous) or response category (i.e., when observed scores are treated as ordered or categorical). Support for *error variances* invariance indicates that the unique variances are equivalent across groups. However, error variance invariance is not generally tested as most methodologists consider this test as overly stringent and unnecessary (Brown 2014; Cheung and Lau 2012).

To date, no studies have examined MI for the IGDS9-SF across different countries. This is despite the recommendation for future studies required to assess whether the measurement and scaling properties of the IGDS9-SF hold across different samples, including those derived from different countries (Pontes & Griffiths 2015). Due to an international push for research on this global phenomenon, the unidimensional factor solution for the instrument’s ratings, indicated in the initial IGDS9-SF psychometric properties study, has been since then replicated in Portuguese, Italian, Lebanese, and Slovenian samples (Monacis, Palo, Griffiths & Sinatra 2016; Pontes & Griffiths 2015, 2016; Pontes, Macur & Griffiths 2016a, b; Wu et al. 2017). Accordingly, findings have generally provided consistent support for the single-factor structure of the IGD construct across the several different populations studied. However, as noted by Pontes and Griffiths (2015, 2016) this does not confirm psychometric equivalence across countries, raising the need for formal tests of MI to be conducted. Addressing this gap in the literature is important because (i) the IGDS9-SF has been gaining popularity in research as it has been extensively used in different studies worldwide for the assessment of IGD prevalence (Monacis, Palo, Griffiths & Sinatra 2016; Pontes & Griffiths 2015, 2016; Pontes, Macur & Griffiths 2016a, b; Wu et al. 2017) and (ii) of its potential use in clinical settings internationally (Pontes & Griffiths 2015).

Given the dearth of MI studies of IGD psychometric assessment tools in general and in particular with regard to the IGDS9-SF, the aim of the present study was to examine the instrument’s MI, based on self-reported ratings of gamers from Australia, the USA, and the UK, for the single-factor model. Prior literature has indicated that cultural differences in general may influence the way addictive behaviors are described and experienced, and that further validation of measurement instruments is required to address discrepancies in conceptualizations and response styles (Gjersing et al. 2010; Landrine and Klonoff 1992). In this context, examining the MI of the IGDS9-SF between these three nations has been prioritized for four compelling reasons. First, a relatively large amount of IGD studies has been conducted in the USA, the UK, and more recently Australia (Kaptsis, King, Delfabbro & Gradisar 2016; Lopez-Fernandez, Kuss, Pontes & Griffiths 2016; Petry et al. 2014). Second, in relation to gaming revenue, these three countries have been ranked within the top 15 countries worldwide (Global Games Market Report 2016). Third, pioneering clinical efforts considering the assessment and treatment of IGD cases has been continuously developed in the USA, the UK, and Australia (see “restart” in the USA, “Video Game Addiction Help” in the UK, the “Network for Internet Investigation and Research” in Australia). Finally, despite their wider perceived cultural similarities (e.g., language, social structure), there are reasons to assume that the IGD diagnostic criteria as assessed by the items of the IGDS9-SF could be addressed differently between participants from the USA, the UK, and Australia (Singelis et al. 1995; Chen and Bouvain 2009).

Cultural variations between the three countries in terms of the dimensions of “vertical” vs. “horizontal” individualism may influence the way in which psychopathology (and thus IGD) is experienced and reported (Stavropoulos, Alexandraki & Motti-Stefanidi 2013; Singelis et al. 1995). The notion of individualism refers to the way in which people perceive the link between individuals and the society (Oyserman et al. 2002). Individualism reflects a tie between the person and the society, where the values of individual goals and self-reliance are emphasized (while collectivism emphasizes more on group interests and values that eventually define the person’s decisions and behaviors; Lee and Wohn 2012). In this context, “vertical” individualism refers to a subtype of individualism where the highlight on individual interests and values is interwoven with inequality among group members (i.e., inequality in opportunities and social welfare). On the contrary, “horizontal” individualism describes a situation where the value of independency is intertwined with the notion of equality between group members (Lee and Wohn 2012; Singelis et al. 1995). Though all three countries are considered individualistic (rather than collectivistic, or focusing on the needs of the community over the individual), their social structures and policies reflect different types of individualism, namely, horizontal (Australia, UK) and vertical (USA) individualism (Lee and Wohn 2012; Singelis et al. 1995). Australia and the UK are considered examples of horizontally individualistic countries, because although they illustrate the autonomous and independent individual, they assume equality between the individual’s self and the self of others. Conversely, the USA, in social policy and cultural practice, assumes independence with a more distinct sense of inequality between individuals, with competition being a key cultural tradition (Lee and Wohn 2012).

The cultural differences within these individualistic countries may influence the way the interpersonal restraint and relationships difficulties associated with how IGD is experienced at the clinical level (and therefore reported) between gamers (Anderson et al. 2016). Among individualists, verticality endorses a sense that inequalities are necessary to preserve hierarchy and reinforces social compliance due to power imbalance, whereas horizontalness decreases it. In that context, acceptance of inequality (vertical individualism) has been associated with a higher tendency to comply with those who are higher in the perceived social hierarchy (i.e., mental health professionals) and a higher inclination to self-blame and guilt (Singelis et al. 1995). Furthermore, with regard to gaming, the distinction in horizontal and vertical individualism (within a single country) has revealed differences in expected outcomes of gaming, with vertically oriented gamers focusing more on ranking and achievement, that may exacerbate their IGD risk (Lee and Wohn 2012; Stetina et al. 2011). Finally, under a broader country-level social context, differences in the experiences of health concerns and behaviors based on equal access to community and healthcare services could influence the awareness of IGD (Clemens et al. 2014). Overall, these differences are envisaged to potentially differentiate Internet gamers’ responses on the IGD9-SF and therefore the instruments psychometric and scaling properties between the three countries (i.e., more pathologized scores, less close to the mean responses in the USA). This hypothesis is in line with studies that have indicated a lack of MI considering measurements of other psychological constructs between Australia, the USA, and the UK (e.g., parental acceptance rejection Gomez & Rohner 2011 and perceived stress reactivity Schlotz et al. 2011).

## The Present Study

Given that the IGD diagnostic framework developed by the American Psychiatric Association in the DSM-5 (American Psychiatric Association 2013) is relatively recent, there is an increasing demand for cross-cultural studies to be carried out using the nine diagnostic criteria for IGD (Király, Griffiths, & Demetrovics 2015; Pontes & Griffiths 2016). This is a much needed step in research if IGD is ever to be recognized as a *bona fide* addictive disorder. In fact, Petry et al. (2014) argued that “establishing the psychometric properties of instruments assessing these nine [IGD] criteria should begin using a cross-cultural perspective*.*” (p.6). In order to contribute to this goal, the present study used three nonprobability normative online samples of Australian, American, and British gamers in order to provide novel cross-cultural insights onto IGD by means of (i) assessing the unidimensionality of the IGDS9-SF and (ii) investigating its MI across the three samples, after controlling for potential gender and age effects.

As for the variables being controlled in the present study (i.e., age and gender), the rationale for this procedure was due to their widely reported associations to IGD score variations (Anderson et al. 2016; Coffey, Carlin, Lynskey, Li, & Patton 2003; Griffiths & Hunt 1998; Hoeft et al. 2008; Pontes et al. 2014). Findings were expected to allow meaningful comparisons of IGD scores between Australian, American, and British gamers using the IGDS9-SF. The present study aimed to respond to repeated requests for measurement consistency considering IGD, illustrated in the international literature. This has important implications for both research and clinical practice, given the increasing utilization of the IGDS9-SF internationally (Van Rooij et al. 2016) as a psychometric assessment tool aligned with the nine IGD diagnostic criteria defined in the DSM-5 (American Psychiatric Association 2013).

## Method

### Participants and Procedure

The Australian (*N* = 171, mean age = 25.72; SD = 5.52; 76.6% males), the American (*N* = 463, mean age = 25.23; SD = 2.76; 57.9% males), and the British (*N* = 281, mean age = 29.49; SD = 9.47; 86.1% males) samples comprised a total of 915 gamers with a relatively even gender split (mean age = 15.54; SD = 0.65; 44.9% males). Data collection procedures were identical between the three countries. Data collection was approved by the Human Research Ethics Committee of the relevant institutions and participants were recruited online. Eligible individuals (adult gamers, permanent residents, or citizens of the countries involved) interested in participating were invited to register with the study via a *SurveyMonkey* link (i.e., Australia and the UK samples) that was advertised across numerous online gaming websites and forums (e.g., http://www.ausmmo.com.au) or *Amazon Mechanical Turk* (AMT) (i.e., USA sample). Participant responses collected via *SurveyMonkey* and AMT are considered appropriate for psychological research that include participants with diverse backgrounds providing reliable responses (Casler et al. 2013; Chandler and Shapiro 2016). The link of the study directed potential participants to the plain language information statement (PLIS). The PLIS explicitly indicated that participation was voluntary and that participants were free to withdraw from the study at any time prior to its completion; any discontinuation of participation, at any point, required no explanation and was without any penalties. Completion and submission of the questionnaire was only possible after participants had provided their consent to partake in the study, and indicated that they understood the nature of the research being conducted. Online data collection was preferred over more traditional paper-and-pencil data collection based on relevant literature recommendations indicating that this method is cost-effective and facilitates accessibility to hard-to-reach groups (i.e., gamers) that were relevant to the present study (Griffiths 2010). Overall, research has shown that online data collection and paper-and-pencil methods are generally equivalent (Pettit 2002; Weigold et al. 2013).

## Measure

### Internet Gaming Disorder Scale–Short-Form

The nine-item IGDS9-SF (Pontes & Griffiths, 2015—see Appendix Table 4) is a short psychometric tool based on the nine core criteria defining IGD as suggested by the DSM-5 (American Psychiatric Association 2013). The IGDS9-SF assesses the severity of IGD and its detrimental effects by examining both online and/or offline gaming activities occurring over a 12-month period. The nine questions comprising the IGDS9-SF are answered using a 5-point scale: 1 (“Never”), 2 (“Rarely”), 3 (“Sometimes”), 4 (“Often”), and 5 (“Very often”). The final score can be obtained by summing participants’ responses to the nine items ranging from 9 to 45, with higher scores being indicative of a higher degree of disordered gaming. Although there is currently no empirical or clinical data supporting the cutoff diagnostic threshold of the IGDS9-SF, a strict diagnostic approach of endorsement of five or more of the nine IGD criteria as assessed by the IGDS9-SF on the basis of answering “Very often” only should be considered, as this approach mirrors the diagnostic framework suggested by the APA in the DSM-5 (American Psychiatric Association 2013). Internal reliability across the three samples in the present study was high and highly comparable across the three countries (see Table 1).

**Table 1:** Descriptive statistics and reliability coefficients for the IGDS-SF9

	Australian sample (*n =* 171)				USA sample (*n =* 463)				UK sample (*n* = 281)			
	*M*	SD	MIC	*α*	*M*	SD	MIC	*α*	*M*	SD	MIC	*α*
IGDS-SF9	18.90	7.63	.50	.90	20.82	7.85	.51	.91	17.99	7.02	.48	.89

*MIC* mean inter-item correlation, *α* Cronbach’s *α* reliability coefficient

### Statistical Analysis

All analyses were conducted with *Mplus* 7 (Muthén and Muthén 2012). First, descriptive analyses for each scale and each sample were conducted. Then, a series of confirmatory factor analyses (CFA) were computed in order to assess the factor structure of the IGDS9-SF across the three samples and its MI accounting for gender and age effects. In brief, this procedure involves comparing progressively more constrained models that test for configural invariance, metric invariance, and scalar invariance (Millsap and Yun-Tein 2004).

It is worth noting that the sequence of analyses described above tests if a given level of invariance is fully satisfied or not. Partial invariance can be explored when full invariance is not supported. When full metric invariance is not established, the researcher can determine the source of the non-invariance by freeing the equality constraints of the factor loadings in the relevant groups sequentially, until a final partial metric invariance model is obtained. The final partial metric invariance model will only have equivalent items with equal loadings constrained equal across the groups. Similarly, if invariance for the initial scalar invariance model is not supported, the source of the non-invariance can be explored by freeing in the relevant groups the equality constraints of the thresholds sequentially, until a final partial scalar invariance model is obtained. The final partial scalar invariance model only includes the invariant indicators with equal intercepts constrained equal across the groups.

To ascertain which factor loadings and intercepts should be unconstrained, three statistical processes were combined in the present study. First, the Sattorra-Bentler (S-B) *X* ^2^ difference test, which is appropriate for the evaluation of model fit differences in nested models (progressively more restricted models), was used to calculate and compare the fit of the different models being tested (Satorra and Bentler 2010). Second, modification indices (MIs) were calculated through *Mplus* and applied (i.e., unconstraining items) for both the loadings and the intercepts based on descending MIs values. Third, the Benjamini-Hochberg multiple testing procedure (Raykov et al. 2013) was implemented in order to locate (i.e., double check) the parameters that violated the MI restrictions. The Benjamini-Hochberg process is considered as the most robust method to assess MI restrictions, as it conducts multiple comparisons to define the false discovery rate (FDR). FDR-controlling procedures are designed to control the expected proportion of “discoveries” (i.e., rejected null hypotheses) that were false (i.e., incorrect rejections) (Benjamini and Hochberg 1995).

## Results

### Data Screening and Preparation

**Fig. 1:**
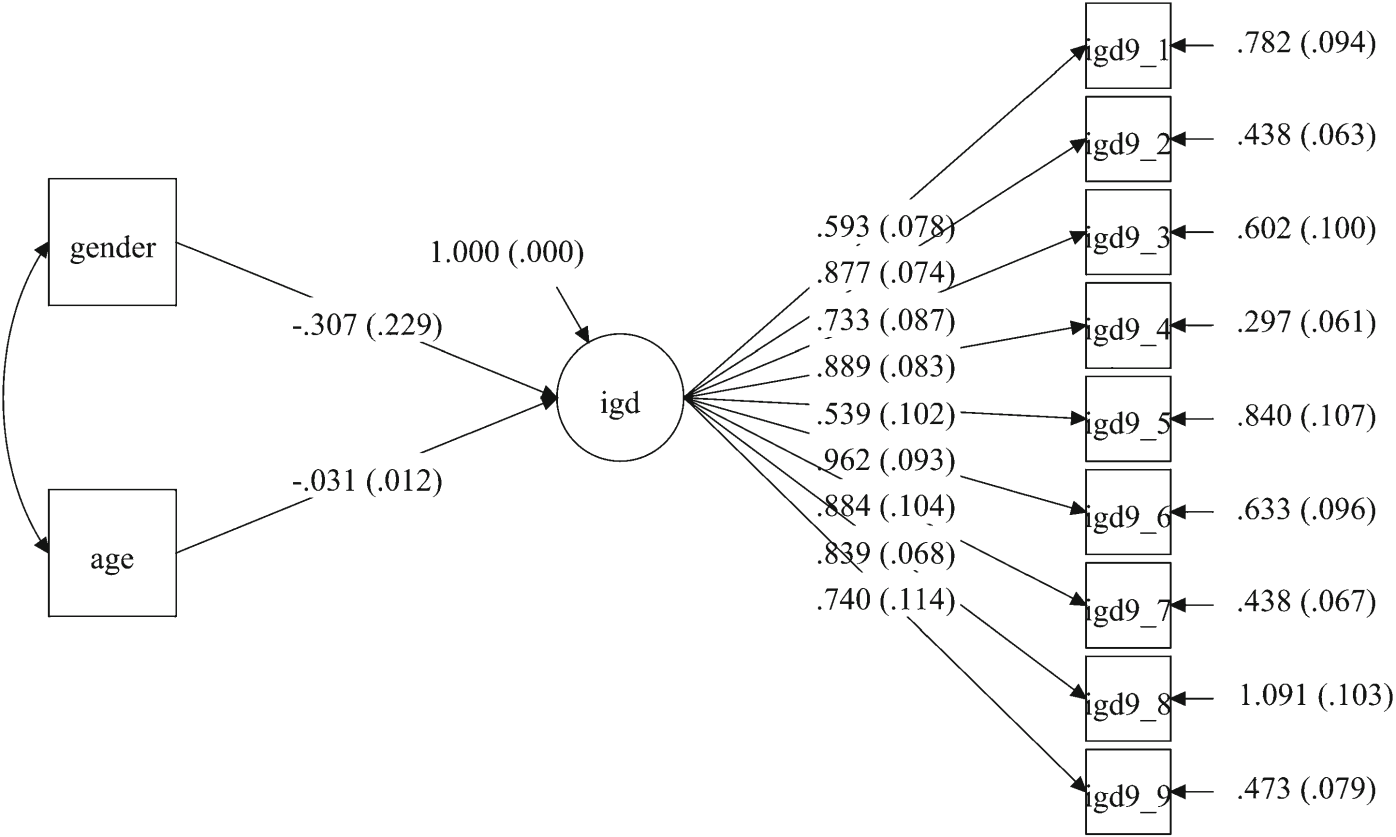
Model for the Australian sample

**Fig. 2:**
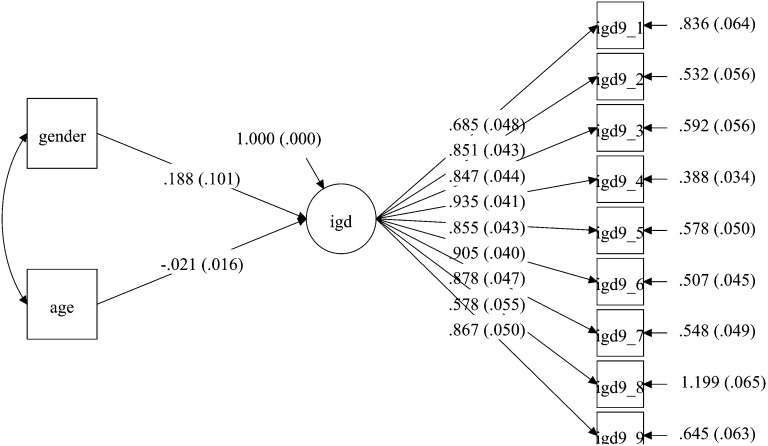
Model for the USA sample

**Fig. 3:**
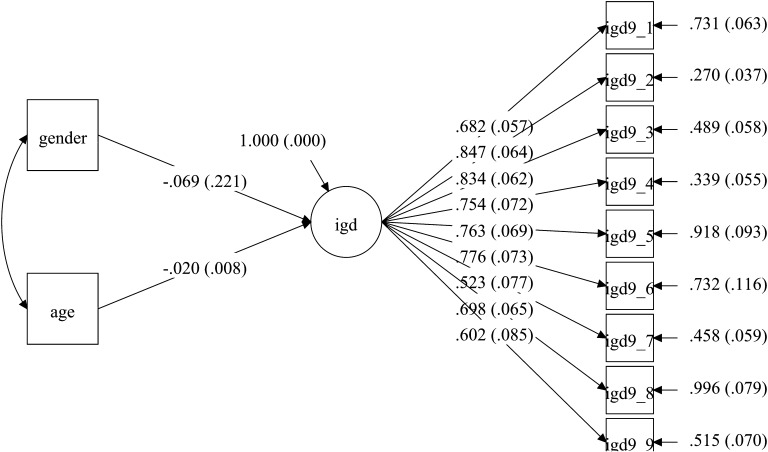
Model for the UK sample

In the Australian sample, item-level missing values ranged from 0 to 6.3% of the sample (Fig. 1). In the USA sample, item-level missing values ranged from 3.2 to 3.8% (Fig. 2). The UK sample did not have any item-level missing values (cases with missing values on all items did not exist in any of the three samples) (Fig. 3). Missing values in the Australian and American samples were addressed with using maximum likelihood imputation (i.e., five times) with all the available variables as predictors. Additionally, screening for multivariate outliers was performed at the item-level through plotting the outlier log-likelihood provided by *Mplus* with the latent variable, which yielded a visual representation of the multivariate outliers. No serious multivariate outliers were found.

### Confirmatory Factor Analysis and MI Outcomes

Table 1 presents descriptive statistics (i.e., means, SDs, mean inter-item correlations, and reliability coefficients) for IGDS9-SF across the three countries. Successive CFAs were computed separately for each country to confirm the one-factor structure of the IGDS9-SF. Overall, the model demonstrated acceptable fit for the Australian (*χ* ^2^ = 85.841, df = 43, *p* = .0001, CFI = 0.936, TLI = 0.920, RMSEA = 0.076, SRMR = 0.051), the American (*χ* ^2^ = 211.81, df = 43, *p*< .0000, CFI = 0.902, TLI = 0.880, RMSEA = 0.092, SRMR = 0.048), and the British (*χ* ^2^ = 115.848, df = 43, *p* < .001, CFI = 0.919, TLI = 0.898, RMSEA = 0.078, SRMR = 0.048) samples. All standardized factor loadings were statistically significant (i.e., *p* < .01) and above .539 for the Australian, above .578 for the American, and above .523 for the British sample.

Following the CFA tests of model fit, the configural invariance (i.e., the unconstrained multigroup) model was computed. Under this process, both *factor loadings* and *intercepts* were unconstrained, thus allowed to differ between groups. The resulting model had an acceptable fit (*χ* ^2^ = 417.705, df = 129, *p* < .001, CFI = .914, TLI = 0.892, RMSEA = 0.086, SRMR = 0.049). Metric invariance (factor loadings fixed, intercepts free) resulted in a drop in fit indices (S-B scaled difference = 62.5554, df = 18, *p* < .001). Holding the intercepts only (i.e., model 3, intercepts fixed and loadings free), and then both factor loadings and intercepts fixed resulted in worsening of fit (S-B scaled difference = 81.0453, df = 18, *p* < .001; Table 2). Those parameters that were non-invariant were located by combining the modification indices and the Benjamini-Hochberg procedure (Table 3). More specifically, factor loadings of items 1, 2, and 5 and intercepts for items 1, 5, 7, and 9 appeared to be non-invariant. Therefore, a final partial invariance model with the above parameters unconstrained was calculated. Partial invariance has lower BIC index than scalar, thus has a better trade-off between model fit and model complexity. Furthermore, the rest fit indices remained adequate, approaching closer those of the configural model.

**Table 2:** Tests of invariance of the IGDS-SF9 questionnaire between Australian, US, and UK gamers with gender and age as covariates

	*X* ^2^	df	*P*	CFI	TLI	RMSEA	BIC	AIC
Configural: loadings + intercepts free	417.705	129	< .0001	.914	.892	.086	21,868.286	21,449.040
Metric: loadings fixed + intercepts free	480.374	147	< .0001	.901	.891	.086	21,825.398	21,492.893
Model 3: loadings free + intercepts fixed	513.403	147	< .0001	.891	.880	.090	21,832.802	21,500.296
Scalar: loadings + intercepts fixed	557.658	165	< .0001	.883	.885	.088	21,784.681	21,538.916
Partial invariance	472.160	151	< .0001	.905	.898	.084	21,786.733	21,473.503

For partial invariance, intercepts for loadings for items 1, 2, and 5 and intercepts for items 1, 5, 7, and 9 were relaxed

**Table 3:** Benjamini-Hochberg procedure: testing intercepts and loadings’ values for IGDS-SF9 invariance between Australian, US, and UK Internet gamers

Model	Parameter relaxed	*X* ^2^ value	df	Satorra-Bentler scaled difference from *M*_0_ df = 1	*P* value
*M*_0_	–	557.66	165	–	–
*M*_1_	*α*_1_	517.54	163	42.7256	< .000000
*M*_2_	*α*_2_	552.32	163	5.0015	.082025
*M*_3_	*α*_3_	555.23	163	1.7108	.425103
*M*_4_	*α*_4_	553.97	163	3.0716	.215288
*M*_5_	*α*_5_	549.83	163	8.6953	.018198
*M*_6_	*α*_6_	555.67	163	0.9569	.619750
*M*_7_	*α*_7_	550.58	163	7.1436	.028105
*M*_8_	*α*_8_	552.69	163	4.4959	.105614
*M*_9_	*α*_9_	549.86	163	7.9560	.018723
*M*_10_	*λ*_1_	541.06	163	16.9811	.000205
*M*_11_	*λ*_2_	547.03	163	14.7031	.000642
*M*_12_	*λ*_3_	554.87	163	2.2315	.448262
*M*_13_	*λ*_4_	554.58	163	0.9432	.623997
*M*_14_	*λ*_5_	548.86	163	8.9891	.011170
*M*_15_	*λ*_6_	553.62	163	2.4154	.298889
*M*_16_	*λ*_7_	554.91	163	5.1647	.075595
*M*_17_	*λ*_8_	552.50	163	4.9948	.082300
*M*_18_	*λ*_9_	557.06	163	2.2576	.320530

*df* degrees of freedom, *α*_*1*_ intercept item l though, *α*_*9*_ intercept item 12, *λ*_*1*_ factor loading item 1 through *λ*_*9*_ factor loading item 9; “parameter relaxed” gives the parameter that is relaxed from the all the loadings and intercepts constraint in model *M*_0_. *P* values refer to the Sattora-Bentler chi-square differences of each of the models test with *M*_0_

## Discussion

The aim of the present study was to evaluate MI of the IGDS9-SF across groups of gamers from Australia, the USA, and the UK accounting for potential confounding effects of gender and age, using the single-factor model, as it has been previously established independently in different countries (i.e., Monacis, Palo, Griffiths & Sinatra 2016; Pontes & Griffiths 2015, 2016; Pontes, Macur & Griffiths 2016a, b; Wu et al. 2017). Based on incremental fit indices values and the S-B *χ* ^2^ difference test, the findings indicated support for the configural invariance and partial support for the metric and scalar invariance. However, lack of full MI has been similarly reported for other psychological constructs measured across Australia, the USA, and the UK (Gomez & Rohner 2011; Schlotz et al. 2011). Notwithstanding this, the support for configural invariance indicates that the single-factor structure of the IGDS9-SF holds invariantly across the different countries compared. The support for partial metric invariance revealed that while the magnitudes of the relationships between the IGDS9-SF items 3, 4, 6, 7, 8, and 9 and the respective IGD latent factor are equivalent (i.e., using the same metric scale) across Australian, American, and British gamers, items 1, 2, and 5 were differentially linked (i.e., unequal associations) to the IGD latent factor across the three groups. Finally, the support for partial scalar invariance indicated that for the same level of the latent IGD trait, individuals across the three groups compared will endorse the same response ratings in items 2, 3, 4, 6, and 8 and different response ratings in items 1, 5, 7, and 9.

With regard to the reported loadings and intercepts inequalities, this finding may be interpreted on the basis of differences considering the cultural dimension of “vertical” individualism across Australia, the USA, and the UK (Stavropoulos, Alexandraki & Motti-Stefanidi 2013; Singelis et al. 1995). Since the USA is considered higher on vertical individualism, the interpersonal restraint and relationships difficulties associated to IGD may be reported differently (Anderson et al. 2016). Following this line of argument, American gamers might be more IGD-vulnerable due to focusing more on game performance and exhibiting lower levels of awareness to addictive behavior due to lower access to mental health and community services, thus presenting with different response patterns in IGDS9-SF (Clemens et al. 2014; Lee and Wohn 2012; Stetina et al. 2011).

Overall, these findings appear to corroborate and compliment studies that have investigated the role of the nine IGD criteria in terms of their diagnostic weight and accuracy. For instance, the study conducted by Király et al. (2017) comprising a sample of 4887 gamers found that although the IGD construct may be effectively measured by a unidimensional factor structure, each criterion accounted for IGD differently in relation to unique stages and severity levels of IGD. More specifically, Király and colleagues (2017) found that the IGD criteria related to “continuation” (IGDS9-SF, item 6), “preoccupation” (IGDS9-SF, item 1), “negative consequences” (IGDS9-SF, item 9), and “escape” (IGDS9-SF, item 8) were associated with lower severity of IGD, while “tolerance” (IGDS9-SF, item 3), “loss of control” (IGDS9-SF, item 4), “giving up other activities” (IGDS9-SF, item 5), and “deception” (IGDS9-SF, item 7) were all associated with more severe levels of IGD. In this context, Rehbein et al. (2015) found that the criteria “giving up other activities” (IGDS9-SF, item 5), “tolerance” (IGDS9-SF, item 3), and “withdrawal symptoms” (IGDS9-SF, item 2) were of key importance for identifying IGD effectively, while Lemmens et al. (2015) reported that “escape” (IGDS9-SF, item 8) did not add further diagnostic accuracy due to lack of specificity. Differences considering the significance of the IGD criteria reported above may be explained on the basis of lack of MI of the IGD measurements used across the different populations studied.

As the Internet continues to integrate into the daily lives of a global community, human-computer interaction will be a domain of continued study and inquiry in cross-cultural research. Addiction studies have demonstrated cross-cultural differences in addiction motivations and expressions (Beyers et al. 2004), and although the IGDS9-SF has been extensively validated in different countries (i.e., UK, Italy, Slovenia, Portugal, and Lebanon) as a theoretically and psychometrically sound instrument, its MI across countries has not previously been confirmed to secure its appropriate use for international comparisons (i.e., Monacis, Palo, Griffiths & Sinatra 2016; Pontes & Griffiths 2015, 2016; Pontes, Macur & Griffiths 2016a, b; Wu et al. 2017). This demonstrates a compelling need for IGD as a globally emerging disorder, with immediate implications for a more valid and reliable interpretation of relevant demographic and prevalence data, and long-term implications for screening and treatment in the clinical contexts cross-culturally.

Notwithstanding the novel insights discussed, the present study includes several limitations. First, this study did not control for factors others than age and gender; therefore, the findings may be confounded by them. One such factor could be related to the unique structural characteristics of the video games played by gamers. Research has found that the structural characteristics of video games may play a different role in the development and maintenance of addictive processes (King, Delfabbro, & Griffiths 2010a, b, 2011; Westwood & Griffiths 2010). For this reason, future research could investigate how the diagnostic accuracy and significance of each IGD criterion may be impacted with regard to different video game structural characteristics, genres, and types (i.e., online and offline) as little is evidence has been produced in this area. Second, all gamers recruited to this study were from the general community, based in Australia, the USA, and the UK. Thus, it is possible that the findings may not be applicable to clinical samples or to different cultural and national groups as further investigation in specific cohorts would be required. Irrespective of these potential limitations, it is hoped the results and information discussed in the present paper will contribute meaningfully towards facilitating further research and to a better understanding of the MI of the IGDS9-SF, clinical practice, and research involving the assessment of IGD across the Australia, the USA, and the UK.

## Conclusion

To summarize, the study’s findings have implications for the utilization of the IGDS9-SF in clinical practice and research across the three countries examined. The invariance findings indicate that the IGDS9-SF viewed in terms of the single-factor model can be justifiably used across groups of gamers completing the measure in Australia, the USA, and the UK. However, IGDS9-SF scores should be compared cautiously across the three countries, given the inequalities in specific factor loadings and intercepts.

## Ethical Statement

All procedures followed for the present study were in accordance with the ethical standards of the responsible committee on human experimentation (institutional and national) and with the Helsinki Declaration of 1975, as revised in 2000 (5). Informed consent was obtained from all participants for being included in the study. No identifying information is included in this article. No animal or human studies were carried out by the authors for this article.

## Appendix

**Table 4:** The Internet Gaming Disorder Scale–Short-Form (IGDS9-SF)

	Never	Rarely	Sometimes	Often	Very often
1. Do you feel preoccupied with your gaming behavior? (Some examples: Do you think about previous gaming activity or anticipate the next gaming session? Do you think gaming has become the dominant activity in your daily life?)	○	○	○	○	○
2. Do you feel more irritability, anxiety, or even sadness when you try to either reduce or stop your gaming activity?	○	○	○	○	○
3. Do you feel the need to spend increasing amount of time engaged gaming in order to achieve satisfaction or pleasure?	○	○	○	○	○
4. Do you systematically fail when trying to control or cease your gaming activity?	○	○	○	○	○
5. Have you lost interests in previous hobbies and other entertainment activities as a result of your engagement with the game?	○	○	○	○	○
6. Have you continued your gaming activity despite knowing it was causing problems between you and other people?	○	○	○	○	○
7. Have you deceived any of your family members, therapists, or others because the amount of your gaming activity?	○	○	○	○	○
8. Do you play in order to temporarily escape or relieve a negative mood (e.g., helplessness, guilt, anxiety)?	○	○	○	○	○
9. Have you jeopardized or lost an important relationship, job, or an educational or career opportunity because of your gaming activity?	○	○	○	○	○
